# New MS lesion segmentation with deep residual attention gate U-Net utilizing 2D slices of 3D MR images

**DOI:** 10.3389/fnins.2022.912000

**Published:** 2022-07-22

**Authors:** Beytullah Sarica, Dursun Zafer Seker

**Affiliations:** ^1^Department of Applied Informatics, Graduate School, Istanbul Technical University, Istanbul, Turkey; ^2^Department of Geomatics Engineering, Faculty of Civil Engineering, Istanbul Technical University, Istanbul, Turkey

**Keywords:** deep residual learning, U-Net, attention gate, convolutional neural networks, multiple sclerosis (MS), MS lesion activity segmentation, lesion activity, MS new lesions segmentation

## Abstract

Multiple sclerosis (MS) is an autoimmune disease that causes lesions in the central nervous system of humans due to demyelinating axons. Magnetic resonance imaging (MRI) is widely used for monitoring and measuring MS lesions. Automated methods for MS lesion segmentation have usually been performed on individual MRI scans. Recently, tracking lesion activity for quantifying and monitoring MS disease progression, especially detecting new lesions, has become an important biomarker. In this study, a unique pipeline with a deep neural network that combines U-Net, attention gate, and residual learning is proposed to perform better new MS lesion segmentation using baseline and follow-up 3D FLAIR MR images. The proposed network has a similar architecture to U-Net and is formed from residual units which facilitate the training of deep networks. Networks with fewer parameters are designed with better performance through the skip connections of U-Net and residual units, which facilitate information propagation without degradation. Attention gates also learn to focus on salient features of the target structures of various sizes and shapes. The MSSEG-2 dataset was used for training and testing the proposed pipeline, and the results were compared with those of other proposed pipelines of the challenge and experts who participated in the same challenge. According to the results over the testing set, the lesion-wise F1 and dice scores were obtained as a mean of 48 and 44.30%. For the no-lesion cases, the number of tested and volume of tested lesions were obtained as a mean of 0.148 and 1.488, respectively. The proposed pipeline outperformed 22 proposed pipelines and ranked 8^th^ in the challenge.

## 1. Introduction

Multiple sclerosis (MS) is an autoimmune disease characterized by demyelinating axons in the central nervous system, resulting in white matter (WM) lesions (Steinman, 1996; Calabresi, 2004). Magnetic resonance imaging (MRI) is widely utilized for various purposes, such as disease diagnosis, patient follow-up, and therapy monitoring. In clinical practice, MRI data can be used to diagnose and assess MS lesions, which helps physicians better understand the natural history of MS (Lladó et al., 2012; Combès et al., 2021). Fluid Attenuated Inversion Recovery (FLAIR) is an MRI technique that provides images in which WM lesions emerge as high-intensity areas, allowing for tracking of the disease progression (Rovira et al., 2015). In particular, this technique facilitates lesion segmentation to acquire quantitative features such as the number and volume of lesions (Roy et al., 2018). Since manual segmentation of such lesions is prone to high interobserver variability and time-consuming processes (Egger et al., 2017; Commowick et al., 2018), accurate automated segmentation methods are required to perform this process (Ma et al., 2022).

The emergence of new lesions or the expansion of existing lesions is referred to as lesion activity (McFarland et al., 1992). The most important biomarker for monitoring inflammatory changes and disease progression in MS is to track lesion activity between two longitudinal MR images (Patti et al., 2015; Combès et al., 2021). Recently, the delineation of new MS lesions on T2/FLAIR by comparing two time-points MRI data has gained attraction. Determination of new lesions has become even more important than identifying the total number and volume of lesions as it allows clinicians to determine whether a given anti-inflammatory disease modifying drug (DMD) is effective for the patient (Moraal et al., 2010). However, detection and delineation of new lesions appearing at the second-time point are particularly challenging and intra- and inter-rater variability are unavoidable due to small and subtle new lesions (McKinley et al., 2020). Therefore, automating the detection of these new lesions will be a significant improvement in assessing the disease activity of a patient.

Recently, deep learning methods, especially those relying on convolutional neural networks (CNNs) (LeCun et al., 2015), have improved the performance of brain lesion segmentation tasks (Akkus et al., 2017); such as brain tumor segmentation (Havaei et al., 2017), brain extraction (Kleesiek et al., 2016), and MS lesion segmentation (Roy et al., 2018; Aslani et al., 2019; Zhang et al., 2019). Most of these methods rely on encoder-decoder networks, taking MRI data as an input and generating a segmentation output for each pixel (Danelakis et al., 2018). Many CNN-based methods and their variations have also been proposed with different input strategies, such as multi-scale (Brosch et al., 2016), multi-branch (Aslani et al., 2019), and cascaded (Valverde et al., 2017) approaches. However, these together with most of the classical methods perform lesion segmentation on a single MRI data. For determining MS lesion activity, classical image processing approaches have been usually preferred such as image differences, intensity-based approaches, and deformation fields (Ganiler et al., 2014; Lesjak et al., 2016; Salem et al., 2018; Köhler et al., 2019). However, some of these approaches have high variability and inconsistency as they use two different segmentation outputs obtained from the baseline and follow-up images to produce the lesion activity (Krüger et al., 2020). To perform better lesion activity segmentation, deep learning approaches relying on CNNs are essential which take these two images as input; however, these methods have been so far limited for the MS lesion activity segmentation. Salem et al. (2020) who used a classical approach in their previous study proposed the first CNN-based longitudinal approach for detecting new T2-w lesions in brain MRI. In their study, intensity- and deformation- based features from two time-points data were incorporated into the proposed network and trained within an end-to-end procedure. Gessert et al. (2020b) have proposed a CNN-based method using two FLAIR images acquired at two different times to detect lesion activity. They used two-path architectures with attention-guided interactions to process two time-points of MRI data. Furthermore, they extended their work to full 4D deep learning using a history of MRI volumes and proposed a 3D ResNet-based multi-encoder-decoder network in which temporal aggregation was performed by convolutional gated recurrent units (convGRUs) for lesion activity segmentation (Gessert et al., 2020a). However, the dataset of these studies consists of MR images from the same scanner, which decreases the generalizability of these methods toward the intensity and texture characteristics variations, which can be inherited if the data is obtained from different scanners. Thus, there is a need for new deep learning approaches to cope with variations problems that may arise through the use of data from multiple scanners as well.

The patch-based and image-based approaches are generally used in CNN-based medical image segmentation (Aslani et al., 2019). Image-based segmentation approaches exploit the global structure information when processing the entire image; however, the patch-based approaches ignore this information due to the small patch sizes. In image-based segmentation, the 3D MRI data is processed either using slice-based or 3D segmentation methods (Brosch et al., 2016; Tseng et al., 2017). In slice-based image segmentation, each 3D MRI is converted into 2D slices along the x, y, and z axes, and then used as an input for deep learning models. After, these processed slices are aggregated to reconstruct a 3D binary output segmentation. In the 3D segmentation, meaningful information from the original 3D images is extracted with 3D kernels in a CNN. However, applying traditional 3D segmentation with a large number of parameters to a small dataset is prone to a high risk of overfitting issues which is a common issue in medical image analysis (Brosch et al., 2016). To address this overfitting issue, several approaches have been proposed such as defining three 2D kernels for each of the three plane orientations around the voxel (Liu et al., 2017; Tetteh et al., 2020); however, these approaches include more parameters for each plane when compared to the slice-based approach (Aslani et al., 2019).

Training deeper neural networks are challenging due to problems such as degradation problem. To solve these issues, He et al. (2016a,b) presented a deeper residual learning framework that uses identity mapping to ease the network training phase. Ronneberger et al. (2015) modified and extended the fully convolutional network (FCN) architecture (Long et al., 2015) to build the U-Net architecture which works with fewer training images and combines feature maps from multiple levels to enhance the segmentation accuracy. U-Net achieves promising results in medical image segmentation by combining low-level features with high-level semantic features. Combinations of U-Net and residual learning were also used for different image segmentation problems, such as road extraction using remote sensing data (Zhang et al., 2018). In addition, the attention gate (AG) model is proposed for automatically learning to focus on more features related to the target structures of various sizes and shapes (Oktay et al., 2018). AG uses high-level features from skip connections and low-level features from an upsampling operation to emphasize important features. This allows the network to focus on the small and subtle lesions appearing in the target MR images.

In this study, an automated segmentation pipeline with a fully convolutional neural network was used to detect and segment the new lesions observed in follow-up images. This study uses images from “Multiple sclerosis new lesions segmentation challenge (MSSEG-2)” ^1^ which consists of 3D FLAIR images acquired from different centers and scanners (1.5T and 3T). Residual units and attention gates are incorporated into the U-Net architecture for the new MS lesion activity task. The slice-based approach was preferred as the input strategy due to the above-mentioned advantages. Slices extracted from these pairs of MR scans were combined by stacking corresponding baseline and follow-up slices into the input channel dimension and then utilized as input values for the proposed model. This study has two major contributions to MRI base lesion activity monitoring. First, it is shown that an encoder-decoder-based architecture, namely U-Net, provided acceptable results in detecting and segmenting the lesion activity. Second, it is demonstrated that using a whole-brain slice approach with the U-Net architecture including residual blocks and modified attention gates significantly improves the segmentation of lesion activity on MRI data acquired from different scanners.

## 2. Materials and methods

### 2.1. Data, preprocessing, and preparation

In this study, a total of 100 patients' MRI data that was associated with MS disease provided by the MSSEG-2 challenge ^2^ was utilized. The voxel size of each MRI data in this dataset varies from 0.5 ×0.5 ×0.5 mm^3^ to 1.2 ×1.2 ×1.2 mm^3^. The dataset was divided into two groups for training and testing. 40 image pairs were used for the training and the remaining were used for testing. For each patient, raw 3D T2/FLAIR MRI pairs were obtained from 15 different MRI scanners at 1.5T and 3T. A rigid registration was applied to these images to bring them into a middle point in which the ground truth data was calculated by the challenge organizers. Thereafter, a consensus delineated ground truth data for the follow-up images were formed by a majority voting among the four experts and validated by a senior expert neuroradiologist.

Data preprocessing is a crucial step for the segmentation task in medical image processing since the raw MRIs may have irrelevant information like non-brain tissues and skulls. Thus, brain extraction followed by N4 bias field correction (Tustison et al., 2010) was performed on these raw 3D images using the Anima MS longitudinal preprocessing script ^3^. Intensity normalization was performed on each 3D MRI scan using the 99^th^ percentile and Kernel Density Estimate (KDE) with the Gaussian kernel similar to one described by Reinhold et al. (2019) and Zhang and Oguz (2020). Then, early fusion was performed on the baseline and follow-up images to produce 2-channel input data allowing the proposed model to obtain temporal features from MRI sequences.

**Figure 1:**
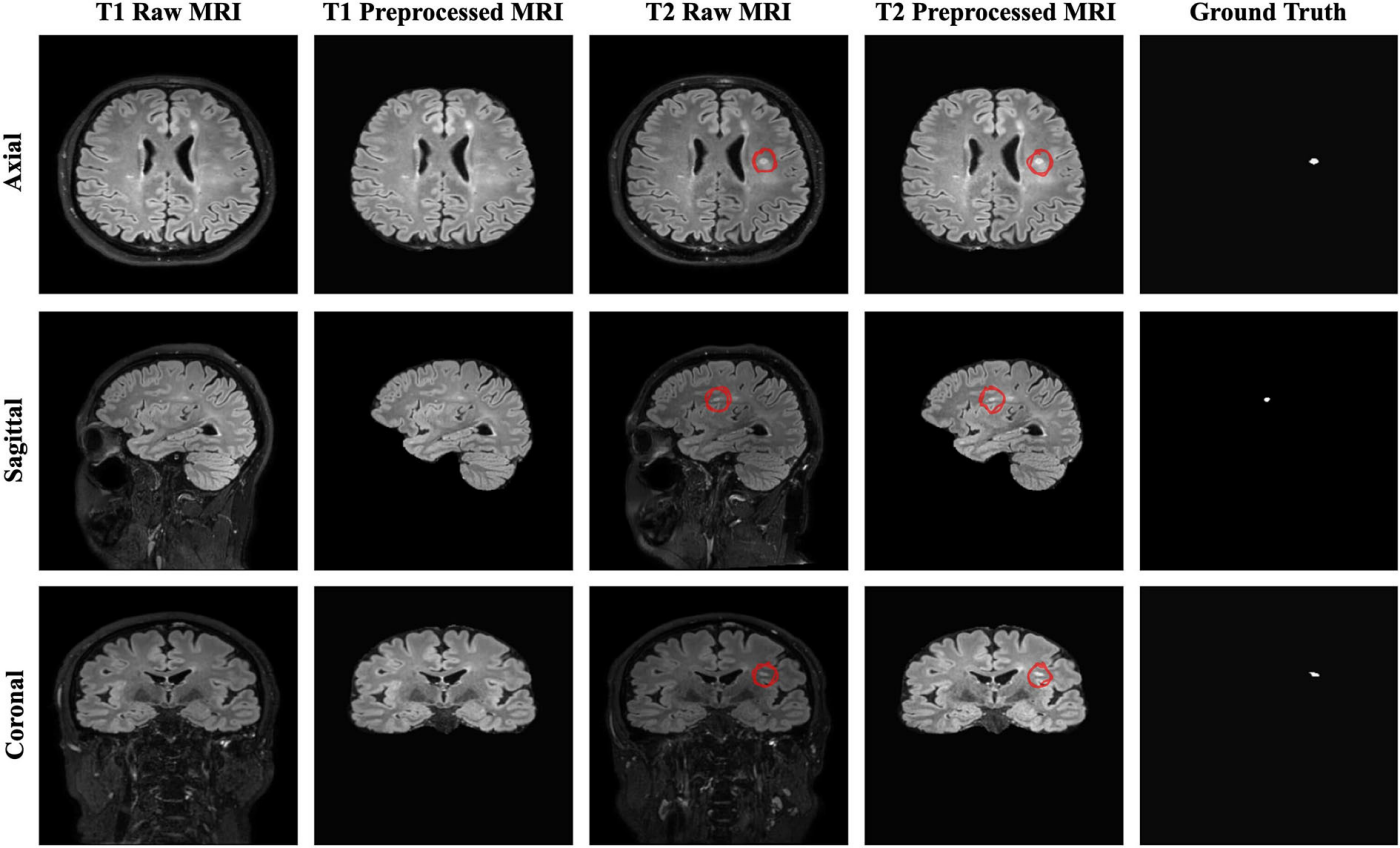
The raw, preprocessed, and delineated mask slices including two-time points for the new MS lesions segmentation task.

The resulting 3D MRI data consists of orthogonal plane orientations which yield three views. From this data, the axial, sagittal, and coronal views along the x, y, and z axes were obtained as 2D slices. Since each generated 2D slice has a different size that depends on the orientation, zero padding was applied to obtain a 512 x 512 slice size for all orientations by centering the brain without affecting the original voxel size. As discussed in detail by Hashemi (2019), zero padding does not deform the patterns in the image and does not affect the network weights during the backpropagation. To restrict excessively unbalanced data and ignore non-informative samples, the slices which have at least one pixel delineated as a new lesion on the follow-up MR images were chosen to create a training subset. As a result, a total of 2,637 2D slices for each time point were derived to be used for training and validation sets. Afterward, the baseline and follow-up images were stacked to generate a 2-channel feature map for each plane orientation. Finally, all 2D stacked slices extracted from all three planes were aggregated to generate a single training input, which allowed to increase training samples and use the contextual information in all directions. Figure 1 shows the raw and preprocessed input data for the two time points dataset with the delineated ground truth data.

### 2.2. Model architecture

#### 2.2.1. U-Net

U-Net, an encoder-decoder network with skip connections, has shown competitive results in the medical field (Ronneberger et al., 2015). This network concatenates features from different levels to enhance segmentation performance. It consists of encoding, bridge, and decoding paths. In the encoding path, the feature map from each layer is downsampled by halving the size to encode the input image into the feature representations. As for the decoding path, the corresponding encoding path which has high-resolution features (semantically low) is combined with the upsampling of the feature maps produced from the lower dimension to better learn representations with the following convolutions. The bridge connects these paths as a transition block. Each block in each layer has two sets of 3 x 3 convolutional layers with a Rectified Linear Unit (ReLU) activation for both downsampling and upsampling operations. The final layer of the U-Net utilizes a 1 x 1 convolution with a sigmoid activation to predict each pixel value ranging from 0 to 1 (Ronneberger et al., 2015). The standard blocks in the U-Net architecture can be replaced with residual units to enhance the model performance.

#### 2.2.2. Residual learning

**Figure 2:**
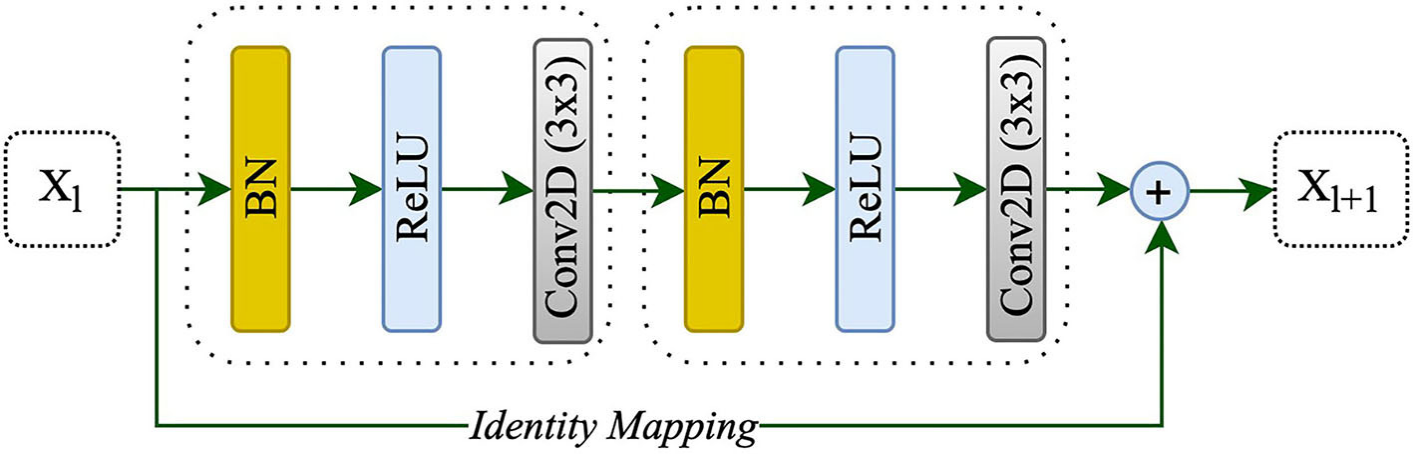
A residual unit with identity mapping. *x*_*l*_ and *x*_*l*+1_ are the input and output of the *l-th* unit, respectively.

Adding more layers to build a deeper neural network could enhance the performance of networks; however, increasing the depth of the network may slow down the training process, perhaps resulting in a degradation problem (He et al., 2016a). Deep residual learning uses several residual blocks together in which an identity mapping is created to handle the performance problem, and also address the degradation problem (He et al., 2016a). The residual unit is comprised of two 3 x 3 convolutional blocks, each with Batch Normalization (BN), a ReLU activation, and a convolutional layer, as well as an identity mapping that combines the input and output of the residual unit. Figure 2 shows the residual unit including identity mapping within the proposed model. Each residual unit is formulated according to He et al. (2016b) as the following:


(1) $$\begin{array}{r} y_{l}=h\left(x_{l}\right)+F\left(x_{l},W_{l}\right) \end{array}$$

(2) $$\begin{array}{r} x_{l+1}=f\left(y_{l}\right) \end{array}$$

where x_*l*_ and x_*l*+1_ are the input and output of the *l*-th unit while F, f, and h indicate the residual function, activation function, and identity mapping, respectively. He et al. (2016b) also recommended a full pre-activation as demonstrated in Figure 2. In this study, a full pre-activation residual unit was used to construct and design the deep residual attention gate U-Net.

#### 2.2.3. Attention gate

**Figure 3:**
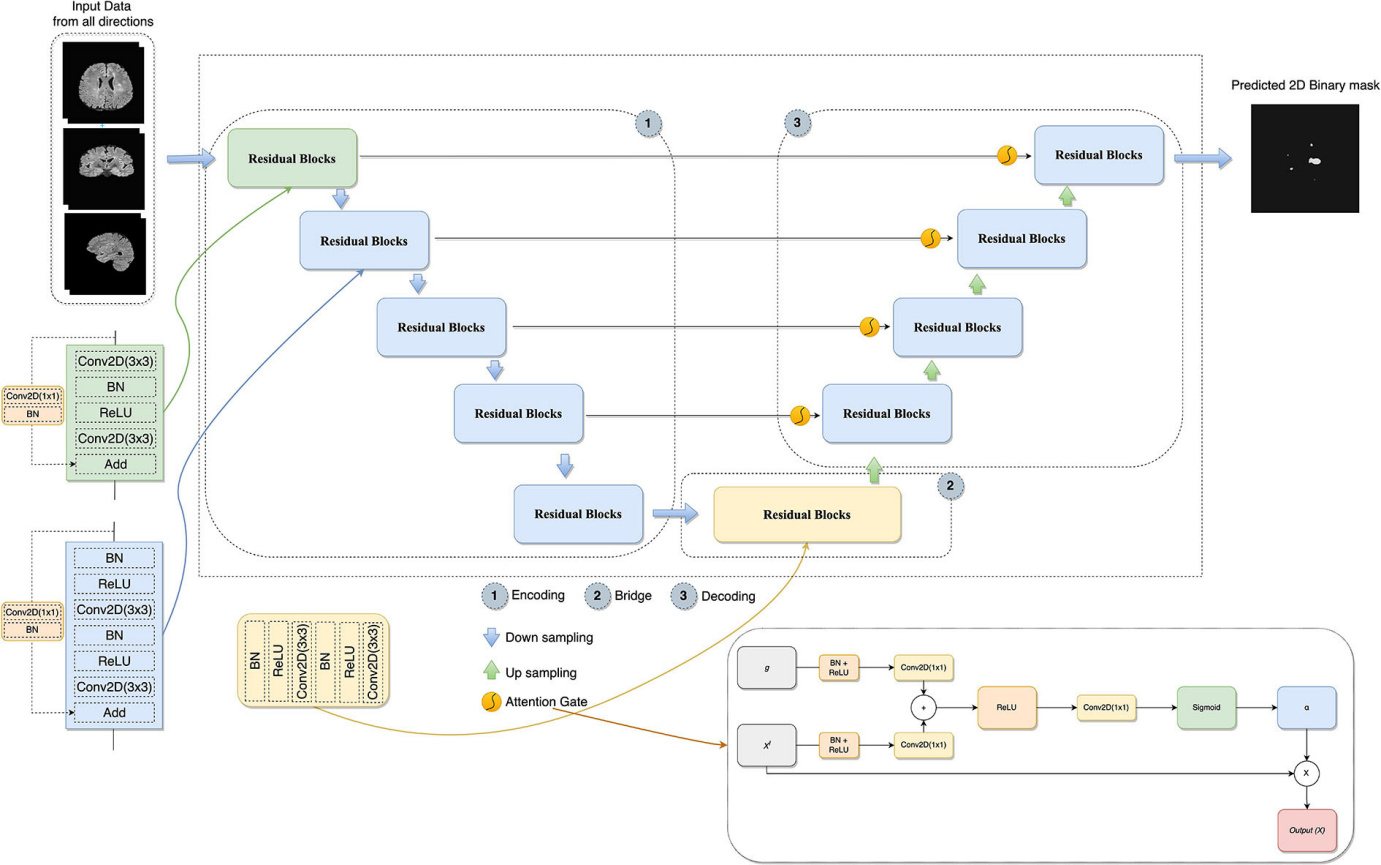
The architecture of the proposed model combines U-Net, residual learning, attention gate, and a slice-based approach. In AG adapted from Oktay et al. (2018), *x*^*l*^ is the input features, α is the attention coefficients used to scale the *x*^*l*^, and *g* collected from a coarser scale is the gating signal which provides contextual information.

Attention gates help the models to focus on learning the salient features beneficial for specific tasks while avoiding unnecessary regions in an input image (Oktay et al., 2018). These are used during concatenating skip connection and upsampling to focus more features related to different sizes and shapes on the target structure. Contextual information (gating) obtained at coarser scales is used to achieve feature selectivity in AGs. Figure 3 shows the overview of the attention gate mechanism.

#### 2.2.4. Deep residual attention gate U-Net

In this study, the combination of U-Net, deep residual learning, and attention gate was proposed for the new MS lesion segmentation task. In this combination, the residual unit will facilitate the network training. Information will be able to propagate without degradation thanks to the skip connections within a residual unit and between low and high levels of the network. Thus, deep neural networks are built with fewer parameters while still achieving a competitive segmentation performance. As such, the standard blocks were replaced with residual blocks in the proposed model. AGs, modified by adding BN and a ReLU activation for both input features before convolutional operations, were added between the corresponding encoding part and the upsampling of features maps produced from the lower level. Thus, allowing the model to learn to focus on salient features of various shapes and sizes. Figure 3 demonstrates the details of the designed network with the input data formed by the axial, sagittal, and coronal views extracted from the baseline and follow-up 3D MRI for the new MS lesion segmentation.

### 2.3. Implementation details

The training set comprised 3D FLAIR images of 40 patients and only 29 had new lesions in their follow-up images. These 29 MR images were divided into the training and validation sets (24 patients for training and 5 patients for validation). To prepare input data, each 3D image was divided into its axial, sagittal, and coronal views. Two-channel input feature data was created using each corresponding 2D slice from both time points as discussed previously. Keras (version=2.4)^4^ and TensorFlow (version 2.4)^5^ libraries were used for the model development in Python language (version 3.7)^6^ (Chollet, 2015; Abadi et al., 2016). The Google Colaboratory, having a Tesla K80 GPU, was used for the training procedure (Bisong, 2019). The proposed model was trained by using the Adam optimizer (Kingma and Ba, 2014), an initial learning rate of 1e-4 (adjusting with patience=10 and factor=0.1 during the training), and a batch size of 8 over 200 epochs, respectively. The validation dice score was also monitored to choose the best model, and model weights were saved based on the best validation dice score during the training. Early stopping (patience=50) was exploited to prevent overfitting as well. Hashemi et al. (2022) used the sum of dice loss with a 1.5 coefficient and binary cross entropy loss as a custom loss function for MS lesion segmentation. Similarly, in this study, a hybrid loss function consisting of binary focal loss and dice loss [dice loss + (1 × binary focal loss)] was employed in order to handle unbalanced labeled data between lesion and background since lesion pixels constitute a minor portion of the whole image. The total loss function is defined as follows:


(3) $$\begin{array}{l} L_{t}=(1-\frac{2gtpr+1}{gt+pr+1})+(1\times (-gt\alpha {\left(1-pr\right)}^{\gamma }\log(pr)\\ \;-(1-gt)\alpha pr^{\gamma }\log(1-pr))) \end{array}$$

where *gt* denotes the ground truth, and *pr* indicates prediction. 0.25 and 2.0 default values were used for the parameters of α and γ, respectively.

**Figure 4:**
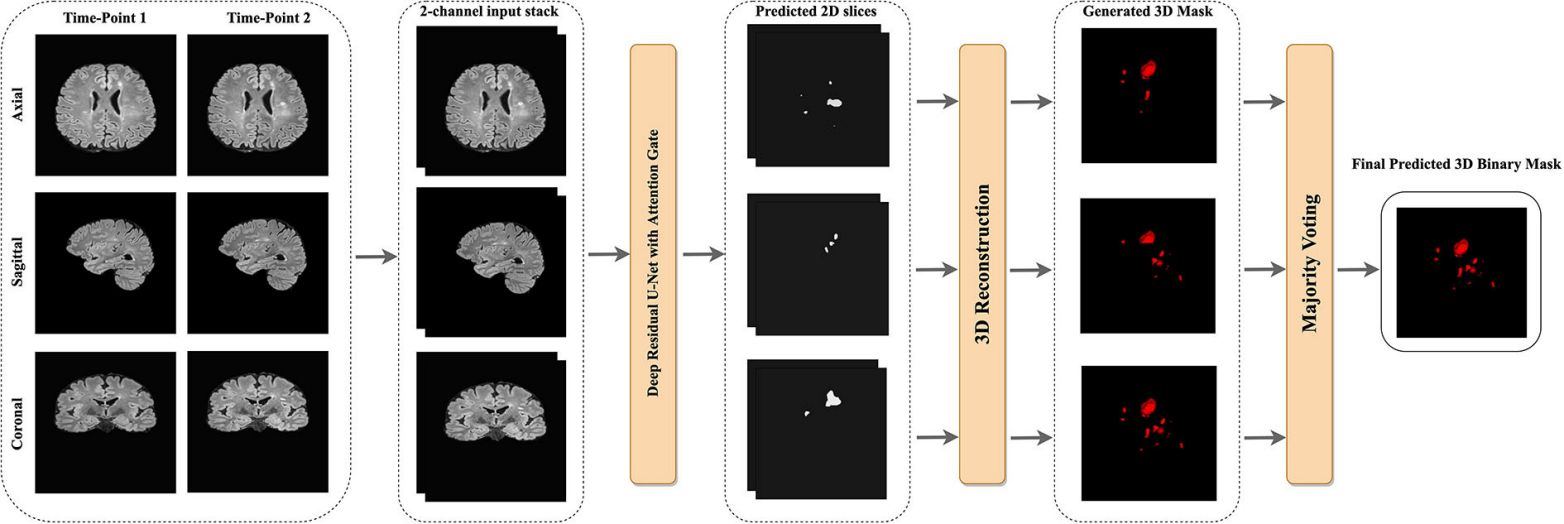
The proposed pipeline of new MS lesions segmentation using a slice-based approach including the majority voting for the final 3D segmentation output using the predicted 2D axial, sagittal, and coronal slices.

Keras data generator was used for performing real-time data augmentation such as vertical flipping, horizontal flipping, random rotation, and shift range to increase the number of training samples. Figure 4 shows the proposed pipeline for new lesion segmentation of MS activity. First, 3D MRIs were converted into their plane orientations along the x, y, and z axes. Then, 2D slices of two-time points were fused together to create a single input training data for the proposed model. Predicted 2D slices based on the axial, sagittal, and coronal views were converted into the 3D binary segmentation output, and then the final output segmentation mask was generated by using the majority voting among 3D binary outputs obtained from each view.

To compare components of the designed network, a testing subset was created from the MSSEG-2 test dataset provided by the challenge organizers. This subset comprised MRI data of 7 patients by considering the different scanners and new lesion loads. Satisfactory results with the MSSEG-2 dataset could not be obtained by the implementation of the original U-Net. Therefore, this implementation was modified with transpose upsampling instead of a simple upsampling operation, and batch normalization to make the neural network more stable. A hybrid loss function, the summation of binary focal and dice losses, was used for all models.

### 2.4. Metrics

#### 2.4.1. Dice similarity coefficient

The segmentation of new lesions was considered one of the two most important evaluation criteria for the challenge. This indicates how many new lesions are precisely overlapped in the ground truth which is also known as the Dice score (Commowick et al., 2018). In other words, the Dice Similarity Coefficient (DSC) is used to measure the similarity of the evaluated segmentation and the ground truth. It is formulated as follows:


(4) $$\begin{array}{r} DSC=\frac{2TP}{2TP+FP+FN} \end{array}$$

where *TP, FP*, and *FN* denote the true positive, false positive, and false negative pixels/voxels, respectively.

#### 2.4.2. F1 score

Another important evaluation criterion was the detection of new lesions. This shows the number of new lesions that are correctly detected or not without considering the precision of their contours. Lesion sensitivity, which is the proportion of the detected lesions in the ground truth, and lesion positive predictive, which is the proportion of TP lesions in the automatic segmentation, were used to compute the F1 score. Lesion sensitivity (*S*) and lesion positive predictive (*P*) can be calculated with the following equations (Commowick et al., 2018):


(5) $$\begin{array}{r} S=\frac{TP_{G}}{M} \end{array}$$

(6) $$\begin{array}{r} P=\frac{TP_{A}}{N} \end{array}$$

where *M* and *N* denote the number of lesions in the ground truth and the automatic segmentation, respectively. *TP*_*G*_ indicates the number of lesions correctly detected by the automatic segmentation among the number of lesions in the ground truth. *TP*_*A*_ denotes the number of lesions correctly detected by the ground truth among the number of lesions in the automatic segmentation. Hereafter, these two metrics can be formulated to calculate the F1 score with the following equation.


(7) $$\begin{array}{r} F_{1}=\frac{2SP}{S+P} \end{array}$$

#### 2.4.3. Metrics for no new lesions

Patients with MS may not have new lesions for their follow-up images. This is usual in clinical cases, and this challenge has also similar cases in both training and test data sets. For example, the testing set is comprised of 28 patients with no new lesions and 32 patients with at least one or more new lesions. The number and volume of new lesions were used as evaluation metrics as well. The volume of new lesions was calculated by multiplying the number of voxels in the segmentation with the voxel volume. A value of zero is the optimal value for these metrics.

#### 2.4.4. Other overlap and surface metrics

Overlap metrics consider the voxel-based overlap of the segmentation output (*A*) and manual annotation mask (*G*) while surface metric computes the average symmetric surface distance. The surface metric considers contours obtained from the segmentation output and manual annotation mask. As described in Commowick et al. (2018), the MSSEG-2 challenge provides a report on the test data set including some of these measures, such as:

- Positive Predictive Value (*PPV*):
(8) $$\begin{array}{r} PPV=\frac{A\cap G}{A} \end{array}$$
- Sensitivity (*S*_*e*_):
(9) $$\begin{array}{r} S_{e}=\frac{A\cap G}{G} \end{array}$$
- Specificity (*S*_*p*_):
(10) $$\begin{array}{r} S_{p}=\frac{B-A\cap G}{B-G} \end{array}$$
  where *B* reveals the entire image.
- Mean Surface Distance (*S*):
(11) $$\begin{array}{r} S=\frac{\sum_{i\in A_{S}}d\left(x_{i},G_{S}\right)+\sum_{j\in G_{S}}d\left(x_{j},A_{S}\right)}{N_{A}+N_{G}} \end{array}$$
  where *d* indicates the minimal Euclidean distance of a point of one surface to the other surface. *N*_*A*_ and *N*_*G*_ reveal the number of points of each surface, respectively.

### 2.5. 3D binary image reconstruction

The slices from each view were used to reconstruct the final 3D binary segmentation output. The 3D binary segmentation was produced by using the 2D predicted slices from each plane orientation. Then, a majority voting was applied to these 3D segmentation outputs to generate the final 3D binary segmentation as shown in Figure 4.

## 3. Results

The MSSEG-2 challenge aims to segment and detect new MS lesions by comparing the baseline and the follow-up 3D FLAIR images of a patient. Twenty four teams with a total number of 30 pipelines participated in this challenge. Deep learning approaches, most of them relying on the U-Net architecture, were proposed by most of the participants, while only one of the teams used a conventional statistical method and the subtraction between two MR images (Commowick et al., 2021). Table 1 shows the average quantitative metric results of some of the methods presented in the challenge, including the results of the experts^7^.

**Table 1:** Prediction results of evaluating the challenge test data set published on the challenge website.

**Methods**	**F1 Score**	**Dice score**	**Number of tested lesions**	**Volume of tested lesions (mm^3^)**
Expert 1	0.712	0.631	0.036	1.453
Expert 3	0.636	0.598	0.000	0.000
Expert 2	0.607	0.536	0.107	3.981
Mediaire-B^*^	**0.541**	0.437	0.536	29.235
Empenn	*0.532*	0.424	0.286	4.258
Mediaire-A	0.525	0.432	0.429	15.908
Expert 4	0.524	0.461	0.036	0.623
LaBRI-IQDA	0.517	*0.500*	1.143	38.486
SNAC	0.514	0.485	0.321	5.726
MedICL	0.500	**0.507**	0.536	12.713
LaBRI-D&E	0.498	0.472	1.964	177.131
**ITU (Ours)**	0.480	0.443	0.148	1.488
New Brain	0.477	0.451	0.786	12.371
LYLE	0.441	0.409	**0.036**	**0.470**
SCAN	0.433	0.403	*0.071*	5.373
Neuropoly-2	0.410	0.409	0.107	*0.498*
SCA-withPriors	0.216	0.224	2.464	302.121
IBBM^+^	0.143	0.155	3.786	123.309

Bold and italic values are the highest and the second-best scores among other proposed methods excluding the experts, respectively. Dice and F1 Score are expected to be a high numerical value while the Number of and Volume of Lesions are expected to be a low numerical value. The Number of and Volume of Lesions metrics are calculated for no new lesion cases. The source data can be accessed at https://doi.org/10.5281/zenodo.5775523. ^*^ and ^+^ indicate the first and last ranks among the participants, respectively. This table is ordered according to the highest to the lowest based on the F1 score.

**Figure 5:**
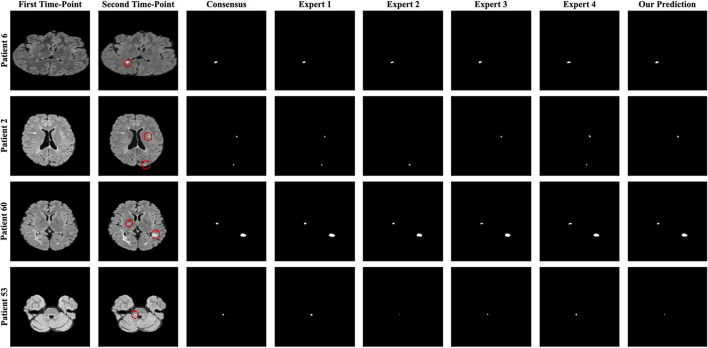
The best and worst performances of the proposed model compared to the consensus and each expert segmentation for F1 and dice scores. A slice of axial view from patients 6 and 2 for the F1 score and patients 60 and 53 for the dice score is presented.

Four metrics were used to evaluate the proposed pipelines for new MS lesion segmentation and detection. The test data set consists of MR images of 60 patients and 32 of them were used for the calculation of the F1 and dice scores due to possessing new lesions at their follow-up images. The remaining patients' data were used for the calculation of the number of tested lesions and volume of tested lesions. According to the challenge results, our proposed pipeline was ranked 8^th^ for F1 and dice scores among the proposed methods. The proposed pipeline produced a mean score of 48% for the F1 score and a mean score of 44.30% for the dice score. For the no-lesion cases, our pipeline was ranked in 5^th^ and 4^th^ places with a mean score of 0.148 and 1.488, respectively for the number of tested and volume of tested lesions. Also, the highest F1 and dice scores including the expert raters were a mean score of 71.20 and 63.10% respectively, which belonged to expert 1. As for the number of tested and volume of tested lesions, the highest score was 0 which belonged to expert 3. On the other hand, the highest F1 and dice scores for the automated methods belonged to teams Mediaire-B and MedICL with a mean score of 54.10 and 50.70%, respectively. The highest score for the number of tested lesions and volume of tested lesions belonged to team LYLE with a mean score of 0.036 and 0.498, respectively. The lowest F1 and dice scores, belonging to the team IBBM, had a mean score of 14.30 and 15.50%, respectively. Figure 5 shows the segmentation performance of the proposed model, consensus, and experts on a slice of an axial view of four patients. As seen in the figure, the proposed model had competitive performance compared to the segmentation output of experts.

The challenge also provides additional metrics discussed in Section 2.4.4 for a complete evaluation although these metrics were not considered for the ranking. The results obtained from some of the proposed methods and experts for additional metrics are given in Table 2. Accordingly, the results of our pipeline with respect to sensitivity, specificity, PPV, and surface distance were a mean score of 0.364, 1.000, 0.675, and 8.548, respectively. Our pipeline had competitive performance compared to experts and other proposed pipelines in some of these metrics. For example, the highest PPV score among experts and proposed methods were a mean of 0.813 and 0.703 for expert 1 and the team LYLE, respectively. Also, the highest score for surface distance belonged to expert 2 and the team LYLE with a mean score of 4.543 and 7.210.

**Table 2:** Prediction results of evaluating the challenge test data set published on the challenge website for other useful metrics.

**Methods**	**Sensitivity**	**Specificity**	**PPV**	**Surface distance**
Expert 1	0.650	1.000	0.707	*5.907*
Mediaire-B	*0.616*	1.000	0.394	8.803
Expert 3	0.589	1.000	0.760	5.990
Expert 2	0.526	1.000	0.813	4.543
MedICL	0.514	1.000	0.556	9.194
Expert 4	0.407	1.000	*0.801*	7.885
Proposed model	0.364	1.000	0.675	8.548
LYLE	0.344	1.000	0.703	7.210
SCAN	0.340	1.000	0.678	8.521
IBBM	0.170	1.000	0.242	24.102

*Bold and italic values are the highest and the second-best scores among some of the proposed methods and the experts, respectively. Sensitivity, Specificity, and PPV are expected to be a high numerical value while Surface Distance is expected to be a low numerical value. The source data can be accessed at https://doi.org/10.5281/zenodo.5775523. This table is ordered according to the highest to the lowest based on the sensitivity score*.

Finally, comparisons between U-Net, U-Net with AGs, U-Net with RUs, U-Net with RUs, and AGs (two types) were realized for the new MS lesion segmentation. The results of U-Net, U-Net + AGs, U-Net + RUs, and U-Net + RUs + AGs are presented in Table 3. As seen in this table, the proposed model achieved the highest dice and F1 scores, a mean score of 58.70 and 61.10%, respectively. U-Net + RUs achieved the highest PPV score, a mean score of 62.40%. Furthermore, this network had fewer training parameters and performed better compared to the U-Net architecture.

**Table 3:** The evaluation results of the proposed method with different components using a subset of the MSSEG-2 test dataset.

**Methods**	**Dice score**	**F1 Score**	**PPV**	**Total parameters**
**U-Net + RUs + AGs**	**0.587**	**0.611**	0.567	4,934,613
U-Net + RUs	0.551	0.441	**0.624**	**4,722,897**
U-Net + AGs	0.505	0.592	0.609	7,947,109
U-Net	0.558	0.490	0.467	7,771,585

*Bold values indicate the highest scores in the columns of dice, F1 score, and PPV while the lowest value in the column of total parameters. This table is ordered according to the highest to the lowest based on the dice score*.

## 4. Discussions

In this study, a deep learning model was developed to handle the problem of identifying new MS lesions using the baseline and the follow-up 3D FLAIR MR images. Activity segmentation particularly for new lesions is a more challenging task compared to lesion segmentation in a single-time MR scan due to small lesion loads. MS lesion segmentation using traditional and deep learning approaches has usually been studied in a single MRI scan in recent years. However, deep learning approaches for MS lesion activity using the baseline and follow-up MR images still remain limited. In most of these studies, the researchers have been using their own datasets making it difficult to compare and reproduce their results with the proposed pipeline. Thus, in this study, comparisons were performed on the automated methods proposed in the challenge. Moreover, comparisons were performed among components used for building the designed network as well. The proposed network, which combines the strengths of U-Net, residual units, and attention gates, has outperformed other methods comprising different combinations of components in terms of dice and F1 scores.

A whole-brain slice-based approach was used as patch-based CNNs lack spatial information about MS lesions due to the patch size limitation (Aslani et al., 2019). The results indicated that the proposed pipeline with this approach had a competitive performance for most measures compared to the other pipelines, as given in Table 1. Segmentation performance of new MS lesions improved significantly when baseline and follow-up MRI scans were stacked in the input channel dimension. Thus, baseline and follow-up scans for each patient were stacked as a two-channel input for the proposed pipeline. Furthermore, attention gates modified with BN and ReLU allowed the model to focus on small and subtle new lesions.

**Figure 6:**
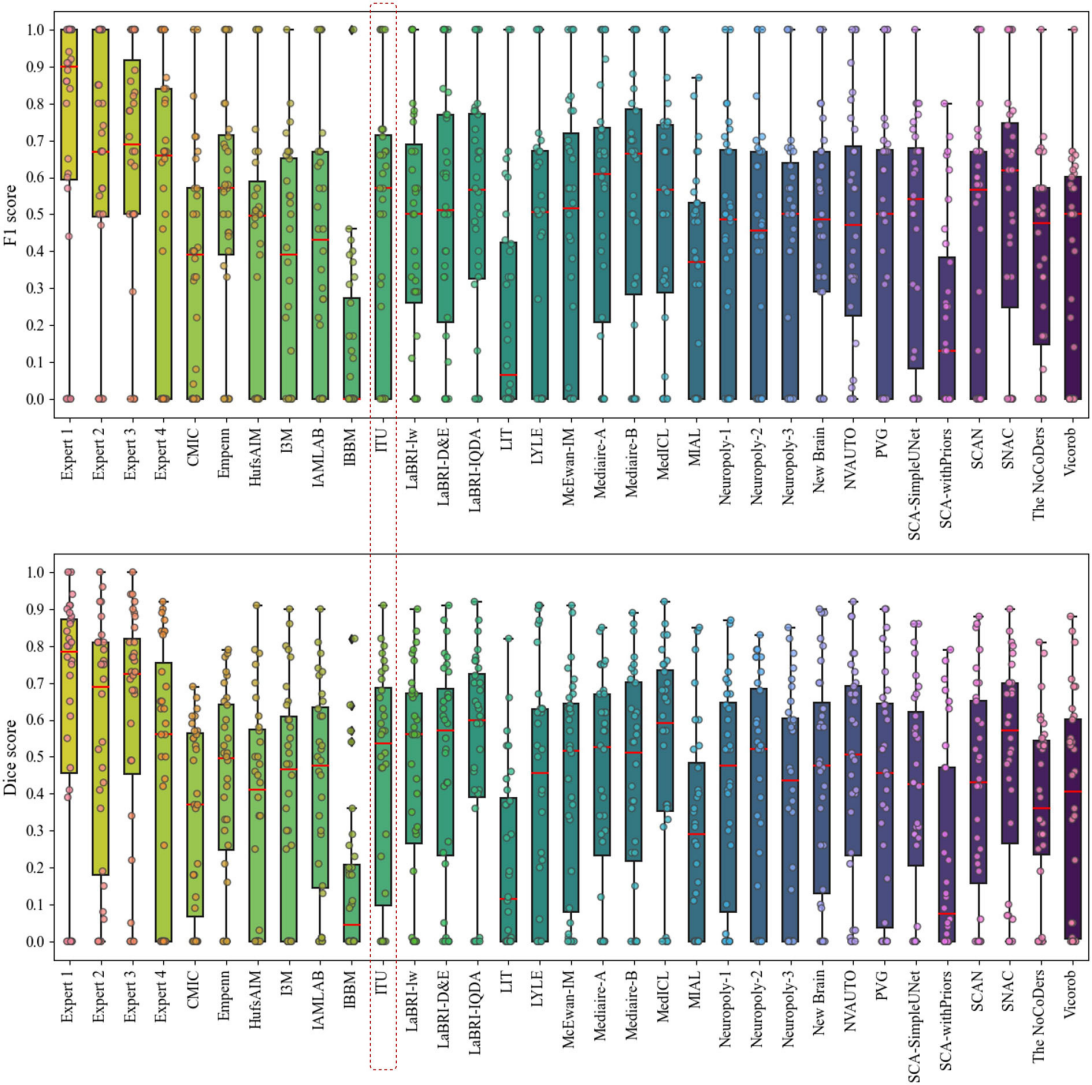
Analysis of differences in detection and segmentation by using F1 and dice scores for each expert and each team that participated in the challenge, respectively.

Figure 6 presents the analysis of differences in detection and segmentation for F1 and dice scores for each expert and each team that participated in the challenge, respectively. The red box highlighted the team performance of this study for these two metrics. According to F1 and dice scores, proposed methods could not reach the expert level; however, some methods were able to outperform experts which revealed varying scores in different patients ^8^. Based on this observation, it was concluded that detection and segmentation of MS new lesions in longitudinal studies is a difficult task even for experts. Therefore, an external reviewer may be needed while analyzing the new lesions with automated methods for the lesion activity.

**Figure 7:**
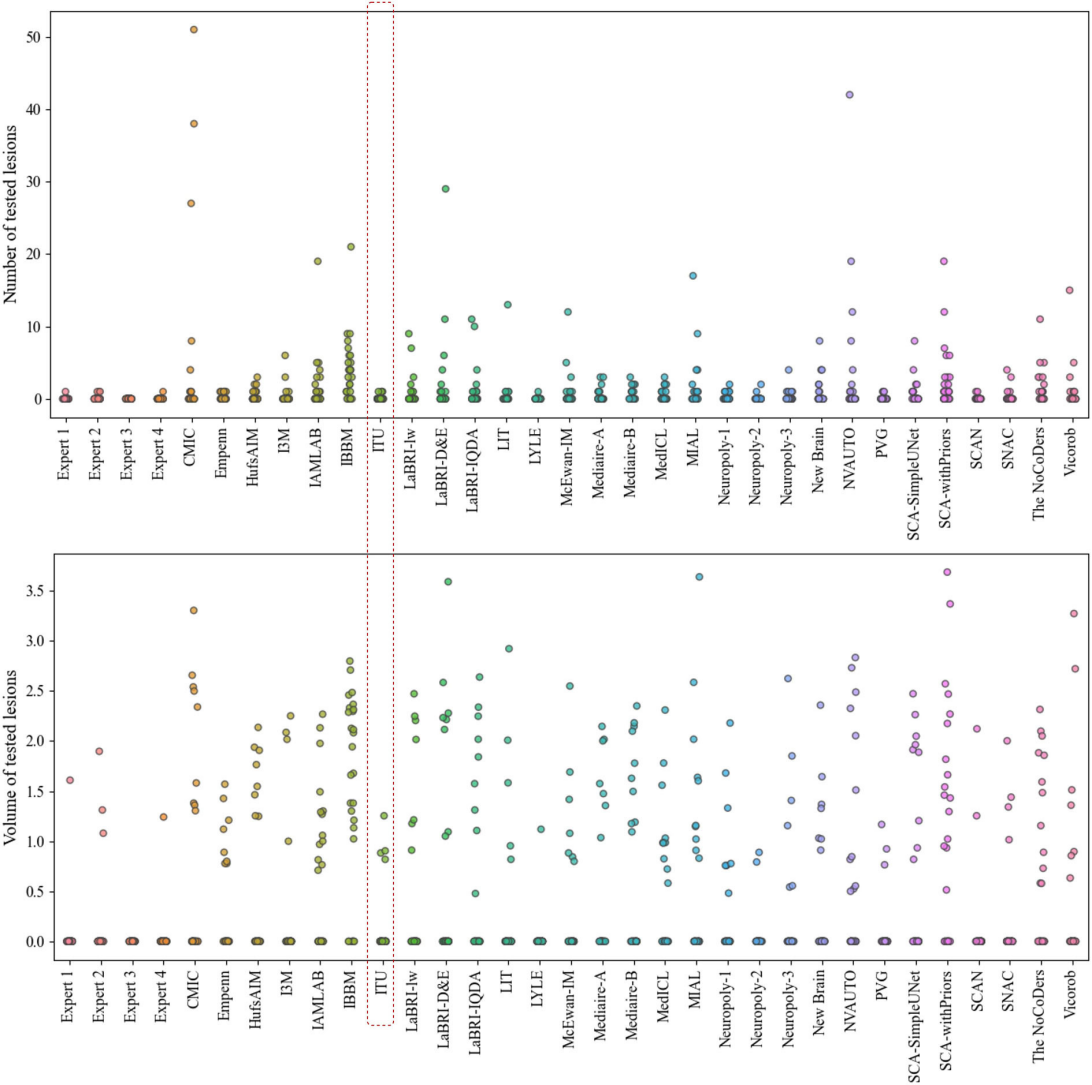
Analysis of the number and volume of lesions detection for each expert and each team that participated in the challenge (The data of volume of tested lesions was scaled by log_10_).

The evaluation metrics for no new lesions are indicated in Figure 7. The number of connected components in automatic segmentation was used to find the number of lesions detected. Also, the volume of lesions detection (mm^3^) was used to evaluate the segmentation performance of both automated and expert delineation outputs. As seen in Figure 7 and Table 1, the proposed pipeline outperformed compared to some of the other proposed methods. The dotted red rectangle highlights the proposed pipeline within this study. Accordingly, some of the proposed methods, including ours, outperformed some experts.

Instead of using a 3D segmentation approach requiring more computational power and learning parameters, the proposed method and the slice-based approach were used together for detecting and segmenting new lesions on the follow-up images. While the appearance of new lesions is of primary interest for the challenge, enlarged or disappearance of MS lesions could be also studied. Different MRI modalities such as T1-and T2-weighted can also be incorporated into the given task to extract more features related to the size or location of new MS lesions even though the FLAIR images reveal lesions as more intense. To achieve a robust automated model for the given task, large datasets from different scanners are needed; however, it is difficult to obtain such datasets.

## 5. Conclusion

In this study, an automated pipeline for new MS lesion segmentation using the baseline and follow-up 3D FLAIR MRI has been designed with a deep learning-based network that fuses the strengths of U-Net, residual learning, and AG. For more accurate segmentation of new MS lesions, this network architecture was designed as a deep encoder-decoder network to enhance the U-Net by replacing plain blocks with residual blocks and adding attention gates. These residual blocks replaced with the plain blocks facilitate the training. Skip connections within both residual units and U-Net facilitates the propagation of information in both forward and backward phases during the training procedure. AGs integrated into the proposed model emphasize important features propagated over skip connections. A hybrid loss function was introduced as the addition of dice loss and 1 × binary focal loss. The input data for the proposed method was prepared by converting 3D scans into their plane orientations of axial, sagittal, and coronal views which yielded 2D slices. Baseline and follow-up slices were stacked to create a two-channel feature mapping for each plane orientation. Then, all slices extracted from all three planes were grouped into a single input to increase training samples and to use the contextual information in all directions. The predicted 2D slices for each view were aggregated using a majority voting to generate the final 3D binary output. Although new MS lesion segmentation and detection pose a difficult problem due to small lesion sizes, the proposed method has achieved comparable segmentation performance compared to the experts and top-ranked automated methods in the challenge. Finding the appropriate data sets and using the existing ones as publicly available will reduce the gap for the data required in these studies and the lack of which is frequently discussed, and will allow different studies to be carried out. This study provides clues about the recent techniques regarding the MS lesion activity segmentation that can be used as a guide for future studies in this field.

## Data availability statement

The dataset used and analyzed in this study can be accessed online at https://portal.fli-iam.irisa.fr/msseg-2/data/.

## Author contributions

BS conducted the experiments and organized the main manuscript. DZS participated in the writing and modifying of the English grammar of the manuscript. Both authors analyzed the results and reviewed the manuscript. All authors contributed to the article and approved the submitted version.

## Conflict of interest

The authors declare that the research was conducted in the absence of any commercial or financial relationships that could be construed as a potential conflict of interest.

## Publisher's note

All claims expressed in this article are solely those of the authors and do not necessarily represent those of their affiliated organizations, or those of the publisher, the editors and the reviewers. Any product that may be evaluated in this article, or claim that may be made by its manufacturer, is not guaranteed or endorsed by the publisher.

## Footnotes

^1^Challenge website: https://portal.fli-iam.irisa.fr/msseg-2/

^2^Challenge Data: https://portal.fli-iam.irisa.fr/msseg-2/data/

^3^Anima scripts: RRID SCR_017072 https://anima.irisa.fr/

^4^ https://keras.io/

^5^ https://www.tensorflow.org/

^6^ https://www.python.org/

^7^Quantitative metrics for all proposed pipeline are available at https://doi.org/10.5281/zenodo.5775523

^8^Evaluation results and analysis slides at https://files.inria.fr/empenn/msseg-2/Challenge_Day_MSSEG2_Results_2021.pdf
